# Detection of Driver Braking Intention Using EEG Signals During Simulated Driving

**DOI:** 10.3390/s19132863

**Published:** 2019-06-27

**Authors:** Trung-Hau Nguyen, Wan-Young Chung

**Affiliations:** Department of Electronic Engineering, Pukyong National University, Busan 48513, Korea

**Keywords:** brain–computer interface (BCI), electroencephalogram (EEG), brain-controlled vehicle, emergency braking intention

## Abstract

In this work, we developed a novel system to detect the braking intention of drivers in emergency situations using electroencephalogram (EEG) signals. The system acquired eight-channel EEG and motion-sensing data from a custom-designed EEG headset during simulated driving. A novel method for accurately labeling the training data during an extremely short period after the onset of an emergency stimulus was introduced. Two types of features, including EEG band power-based and autoregressive (AR)-based, were investigated. It turned out that the AR-based feature in combination with artificial neural network classifier provided better detection accuracy of the system. Experimental results for ten subjects indicated that the proposed system could detect the emergency braking intention approximately 600 ms before the onset of the executed braking event, with high accuracy of 91%. Thus, the proposed system demonstrated the feasibility of developing a brain-controlled vehicle for real-world applications.

## 1. Introduction

In the past decade, brain–computer interface (BCI) has emerged as a potential technology for decoding neural activities into commands by using electroencephalogram (EEG) signals. This new technology allows paralyzed people to communicate using their minds. As an alternative to the conventional communication pathways (i.e., using peripheral nerves and muscles), BCI establishes a direct communication pathway between a human and a machine [1,2,3]. Many researchers have focused on developing BCI systems to improve the quality of life of disabled people. BCI-based wheelchairs were introduced in [4,5]. In other studies [6,7,8], researchers used EEG signals for communication and rehabilitation of lower-limbs or arms. Recently, EEG signals have been used to develop driving-assistance systems. Most of these studies have focused on using EEG signals to monitor physical conditions or mental states of the driver, e.g., drowsiness [9,10] or mental workload [11,12], as an effort to reduce traffic accidents.

Recent technologies have adopted external sensors (i.e., radar, laser, camera) to detect potential car crashes. Consequently, it allows modern cars to enter the emergency mode as soon as the driver presses the gas pedal. However, due to the limited time available in critical situations, time is considered as the first priority. Pressing the gas pedal is the final action of the driver in an emergency braking situation. Fortunately, it is possible to detect the braking intention of a driver earlier by using behavioral data of the driver, such as the steering angle, foot position, and head movements [13,14,15,16]. According to [17], an upcoming emergency situation was detected during simulated driving using event-related potential (ERP) features. The study demonstrated that neurophysiological signs associated with emergency braking occur approximately 130 ms before the onset of the executed braking event. The results suggested the feasibility of detecting the braking intention during simulated driving using EEG signals. Early detection of the braking intention might reduce the risk of car crashes related to late braking or machine failure. To our knowledge, few studies on the detection of the emergency braking intention using EEG signals have been performed. In [18], experimental results for two subjects indicated the feasibility of detecting the braking intention in an emergency situation. However, the detection accuracy was low (76.4%), raising concerns about the reliability of the system for real-world driving.

The objective of this study is to improve the system performance and reliability for detecting the emergency braking intention. A novel combination of autoregressive (AR) feature extraction and artificial neural network (ANN) was introduced. With the combination of the AR extractor and neural network classifier, we obtained meaningful information from patterned EEG data without quantification or visualization, in contrast to methods using the readiness potential, event-related desynchronization (ERD), and event-related potentials (ERPs) features [19]. Additionally, we developed a method to automatically and accurately label data during the training process based on the motion sensing of the braking foot. The detailed analysis performed in this study provides insights into human neural activities during emergency situations.

## 2. Materials and Methods

### 2.1. EEG Headset Design

In the current headset design, we use a dry sensor instead of a wet one owing to its advantages with regard to the contact quality through hair and reusability. The high output impedance of the sensor makes it very sensitive to noise [20]. Therefore, the potential from each electrode is first passed to an active-shield circuit to minimize the electromagnetic interference from external sources. The detailed design of the active sensor is presented in [21].

**Figure 1:**
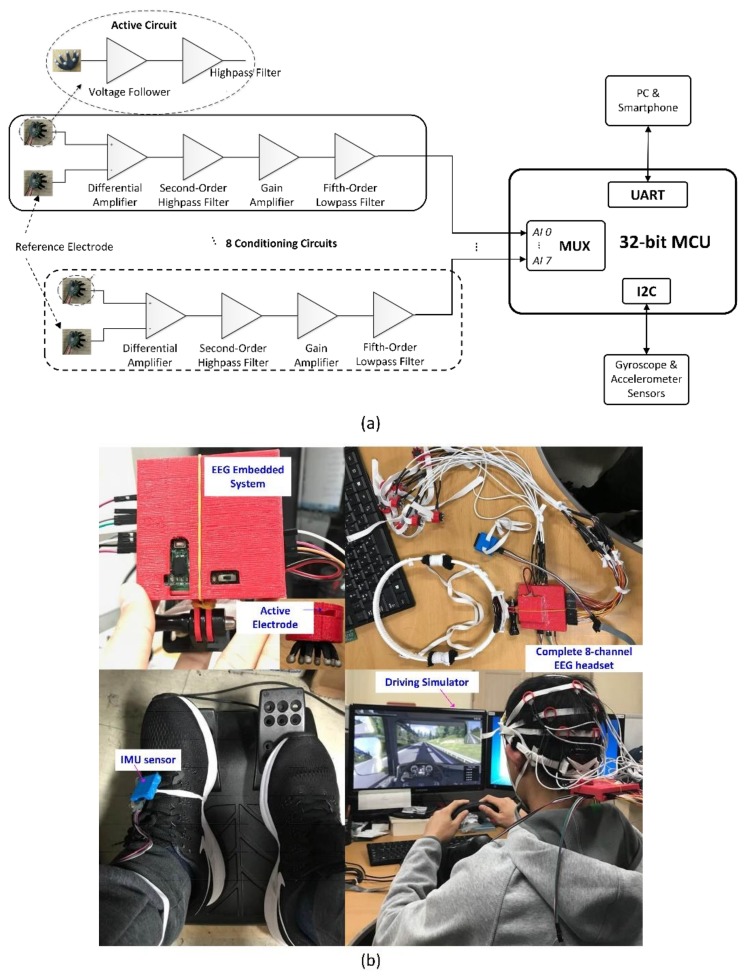
Electroencephalogram (EEG) headset: (**a**) schematic of the 8-channel EEG circuit with dry active electrodes, (**b**) photographs of complete 8-channel EEG headset with 3-D printed flexible cap and the experimental setup for the virtual driving environment.

Figure 1a shows the schematic of the 8-channel EEG circuit. The current headset acquires EEG signals from eight channels (F3, F4, C3, C4, P3, P4, O1, and O2) according to the International 10–20 System. Cz serves as the reference electrode. The differential potential (active channel − reference channel) from each pair of electrodes is amplified and then filtered with a second-order high-pass filter. The signal is then amplified by the main amplifier to meet the analog-to-digital converter (ADC) range (0–3.3 VDC). To avoid aliasing, the signals are filtered with a fifth-order low-pass filter before entering the ADC of the microcontroller (MCU) via a multiplexer. There are eight conditional circuits in total corresponding to the eight EEG channels. To measure multiple analog signals, we employ a multichannel ADC reading procedure using the MCU built-in multiplexer. The sampling frequency is set at 128 Hz using built-in Timer 2 (interval of 7.8 ms). On the other hand, Timer 1 is used to trigger the sampling event of each channel.

In this work, an inertial measurement unit (IMU) sensor (accelerometer and gyroscope) is used to detect the movement of the foot of the driver during emergency braking events. Two such sensors are integrated into one board and communicate with the MCU via an I2C communication bus. The data package containing 8-channel EEG data and IMU sensor data is transferred to a personal computer (PC) via a universal asynchronous receiver/transmitter (UART). Photograph of the proposed EEG headset is shown in Figure 1b. The body of our EEG headset is fully fabricated with a flexible material using the three-dimensional (3D) printing technique. The EEG main embedded system is protected by a 3-D printed housing. The active sensor is fixed in a small holder, which helps to maintain good contact between the electrode and the scalp skin. The IMU sensor is placed inside a housing attached to the foot by an elastic rope.

### 2.2. Experimental Procedure

Eight male and two female subjects (aged from 24–32) participated in the experiment. All the participants were physically healthy and had no visual impairment. The subjects were seated on a comfortable chair in a simulated driving station (Figure 1b). They were asked to drive for approximately 20 min while wearing the proposed EEG headset, using the SCANIA Truck Driving Simulator software. In the driving software, “reaction test” was selected as the driving scenario. The emergency braking situation was simulated by the sudden appearance of a pedestrian or animal crossing the street at a predefined short distance (by the software) from the vehicle. The subject was asked to maintain the car speed at approximately 100 km/h. The reaction time of the drivers was automatically monitored by the software according to the time interval between the emergency-stimulus onset and the execution of the braking event. Prior to the experiment, all participants were instructed to perform a driving trial to familiarize them with the simulated driving tasks. All subjects were asked to be completely focused on the driving tasks, similar to the case of real-life driving.

### 2.3. System Structure and Data Preprocessing

**Figure 2:**
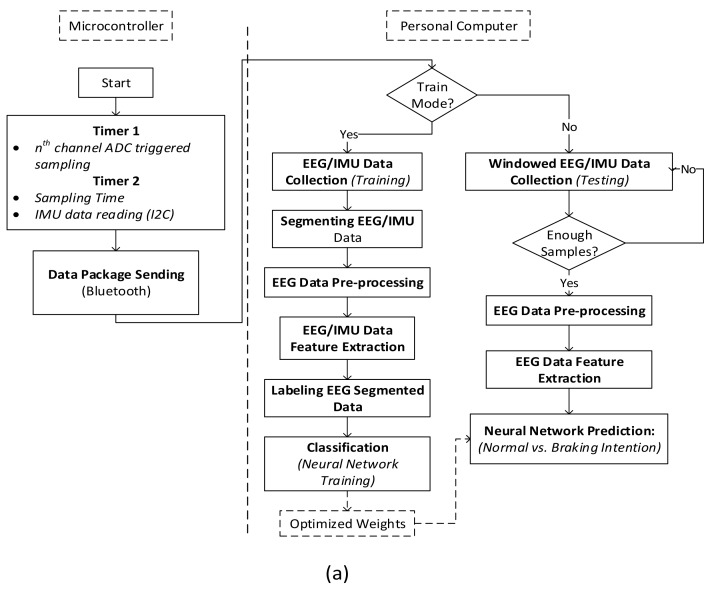

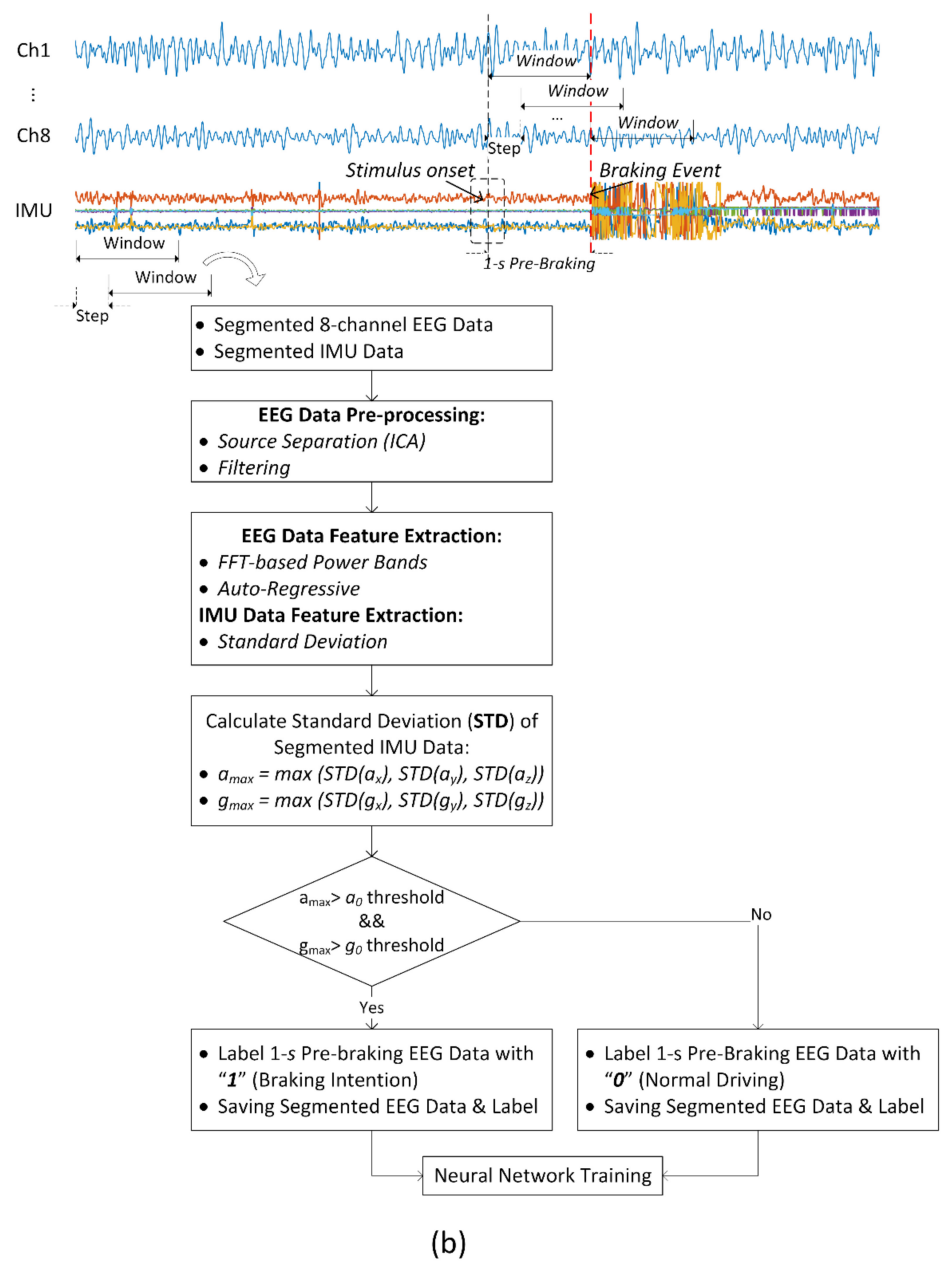
(**a**) Flowchart of the EEG-based braking-intention detection system, (**b**) flowchart of the labeling process for EEG data using the inertial measurement unit (IMU) sensor.

Figure 2a shows the flowchart of the whole system. It consists of training mode and testing mode. In the training mode, 20-min 8-channel EEG and IMU data are captured during the simulated driving task. In contrast to [22], the training data are collected by sliding the window along the data length, in the same manner as online testing. The analyzed window is set as 1 s, and the step size is varied to investigate its effects on the system performance. The IMU data are segmented similarly to the EEG data. To pre-process the data, the segmented 8-channel EEG data are band-pass filtered from 1 to 60 Hz that covers EEG wave bands of interest (delta, theta, alpha, beta, and gamma) as well as to remove power line noise (i.e., 60 Hz). Independent component analysis (ICA) is performed to separate other unexpected noises. In particular, one second of the 8-channel EEG data can be expressed as:

***X***(*t*) = [*x*_1_(*t*), *x*_2_(*t*),…, *x*_8_(*t*)]^*T*^ (1)

where *x_i_*(*t*) represents the EEG data recorded from the *i^th^* channel at time *t*.

The independent components ***Y***(*t*) = [*y*_1_(*t*), *y*_2_(*t*),…, *y*_8_(*t*)] are obtained as:

***Y***(*t*) = ***WX***(*t*) (2)

where ***W*** is the un-mixing matrix, which is calculated according to the infomax algorithm [23]. To remove motion artifacts (blinking, head movement), we first calculate approximate entropy for all independent components. Then, the components associated with these artifacts are eliminated using thresholds.

In the testing mode, the EEG data are analyzed every 1 s. In particular, a segment of EEG data which size is equal to that corresponding to the step size (8, 16, and 32 samples for 62.5-ms, 125-ms, and 250-ms step-sizes, respectively) are added to the buffer for real-time analysis. The remaining steps, including pre-processing and feature extraction, are the same as those in the training mode. Finally, a well-trained neural-network classifier is employed to make predictions on the new segmented EEG data (Figure 2a).

### 2.4. Feature Extraction

Two types of features are investigated in this study. The first feature is based on the frequency domain and is obtained using the fast Fourier transform (FFT), which is a popular nonparametric approach for computing the power spectral density (PSD) of a signal. In particular, a 512-point FFT is performed on the 1-s segmented EEG data, resulting in a frequency resolution of 128/512 ≈ 0.25 Hz. Accordingly, for all eight channels, we compare the difference of the relative power spectra of each EEG band between two situations (normal driving vs. braking intention): the delta band (1–4 Hz), theta band (4–8 Hz), alpha band (8–14 Hz), beta band (14–30 Hz), and gamma band (30–60 Hz). The relative power of each EEG band is computed as follows:

(3) $$rPB_{i\;}=\frac{PB_{i}}{\sum_{i=1}^{5}PB_{i}}$$

where $PB_{i}$ and $rPB_{i}$ represent the absolute and relative EEG power of the *i^th^* band (*i* = 1, …, 5, corresponding to the delta, beta, alpha, theta, and gamma bands), respectively.

The second feature is based on the AR model. As an alternative to the PSD-based feature, AR modeling has been widely used in recent EEG studies as a feature-extraction technique. Compared with the PSD-based approach, AR modeling can provide good spectral resolution for short data segments and accurately detect abrupt changes in the spectra [24]. In previous studies, AR modeling exhibited better performance than the PSD-based method [25,26] for EEG classification. There are several methods for estimating the AR coefficients, such as Burg method, least-squares approach, and Yule-Walker approach. In this study, the AR models are derived via the popular Burg method, which allows high-resolution spectral estimation from short data segments. However, this method is sensitive to the order of the model. Hence, we also investigate the effect of the AR order number on the system performance. The best AR order number with regard to the classification accuracy is selected for the proposed system. The AR model of the EEG data from each channel can be expressed as follows:

(4) $$X_{t}=c+\overset{p}{\underset{i=1}{\sum }}\varphi_{i}X_{t-i}+\varepsilon_{t}$$

where $X_{t}$ represents the single-channel EEG data at time *t*, p is the order number, *c* is a constant, $\varepsilon_{t}$ represents the white noise with zero mean and finite variance, and $\varphi_{i}$ represents the AR coefficients to be estimated.

**Figure 3:**
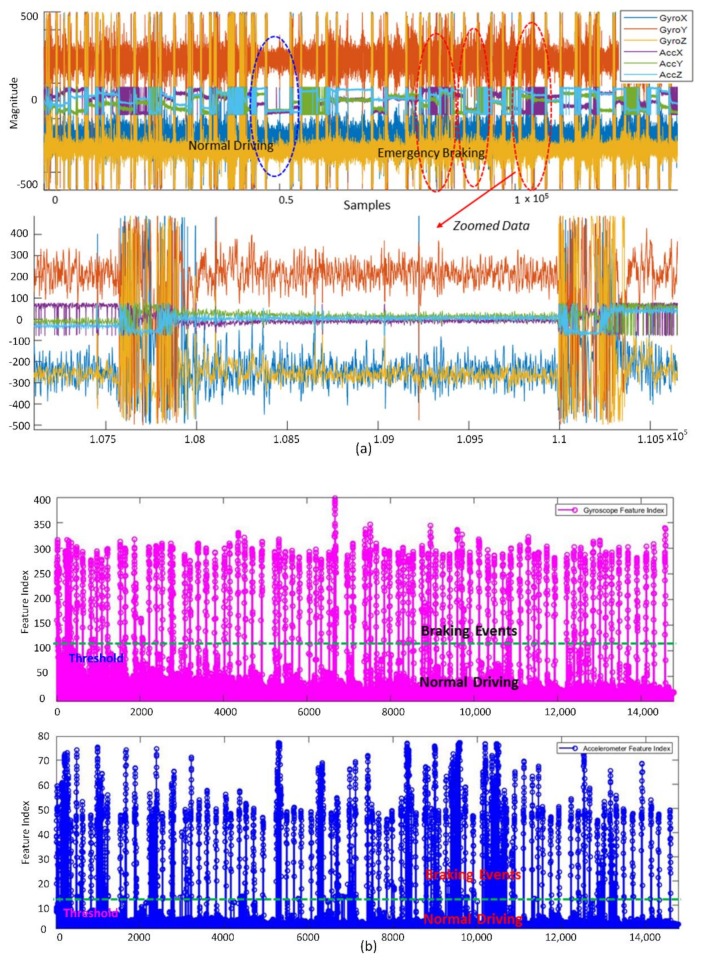

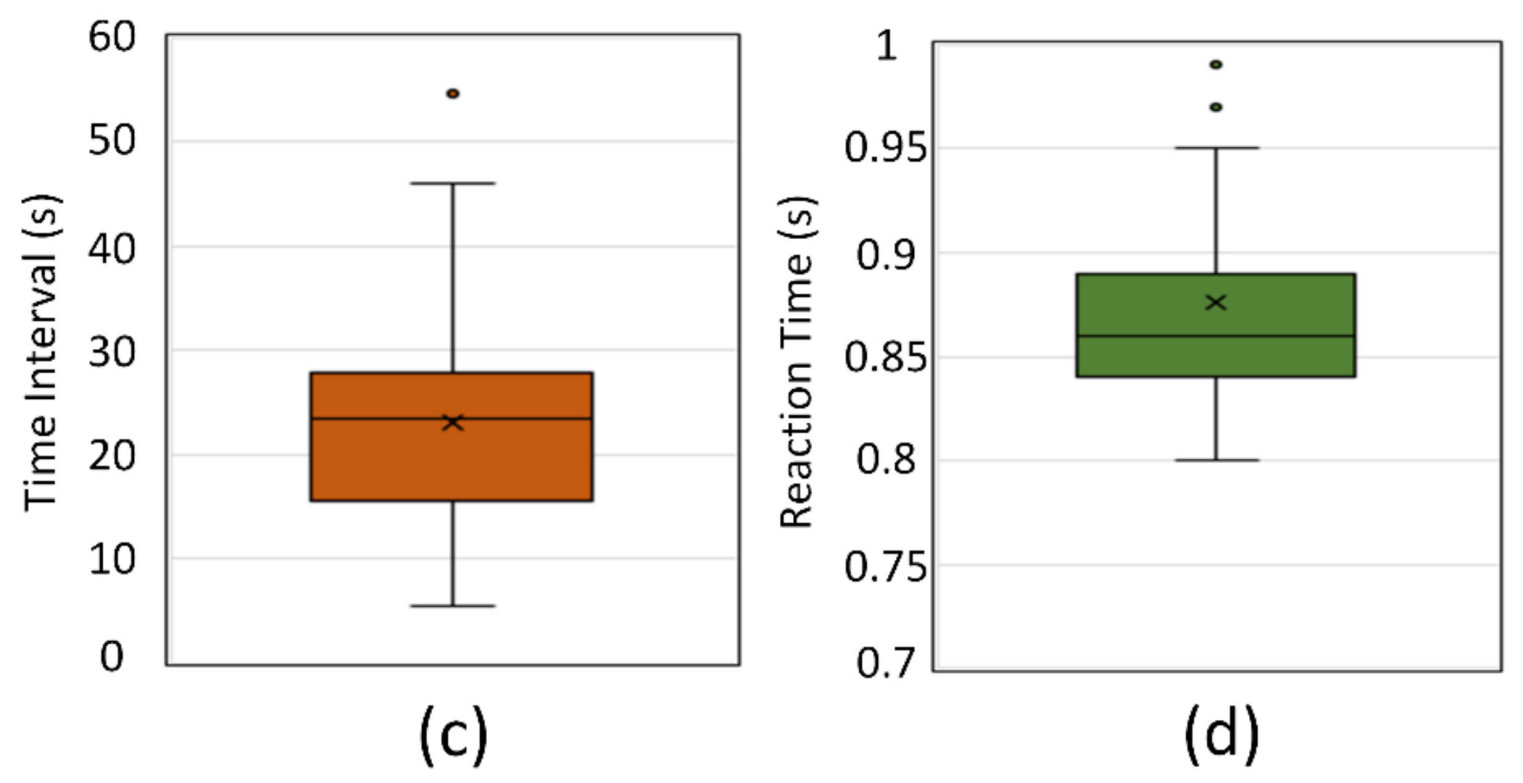
(**a**) Accelerometer and gyroscope data for 10 min of driving, with indicators for normal driving and emergency braking events; (**b**) features of the standard deviation of the windowed data: gyroscope features (top) and accelerometer features (bottom); (**c**) distribution of the time interval between two consecutive braking events generated by the driving simulator; (**d**) reaction time measured by the driving simulator.

In several previous studies [18,22], there was a lack of information regarding how to accurately label the training data in cases where time should be carefully considered. According to [27], ERD starts before the actual movement approximately 150 ms before the electromyography (EMG) onset. Another study [17] demonstrated that neurophysiological correlates of emergency braking occur approximately 130 ms earlier than corresponding behavioral responses related to the actual braking event. Furthermore, according to [22], emergency braking intentions can be detected approximately 420 ms after the onset of an emergency stimulus. Therefore, in this study, we explore the neurophysiological response within 1 s before the executed braking event. Figure 2b shows the procedure of how the data are captured and labeled using the IMU sensor. The braking event can be captured in real time using threshold based on the standard deviation of the data. Once the braking time point is determined, the 1-s EEG data prior to it are labeled as the braking-event samples. The pre-braking period is defined as the time interval before the onset of the executed braking event, in which the data samples are examined to detect the braking intention. According to the simulated driving result, the average reaction time measured by the software is approximately 876 ms (after the onset of the emergency braking stimulus) (Figure 3d). Considering this result, we investigate five pre-braking periods 200, 400, 600, 800, and 1000 ms to estimate the most appropriate neurophysiological response time to the emergency situation. All the training samples within 5 s after the onset of the executed braking event are discarded owing to the instability of the signals immediately after the onset of the emergency situation (i.e., the subject might experience mental suffering due to the dangerous situation). On the other hand, normal-driving samples are obtained during normal driving without an emergency situation based on the small variation of the IMU data.

### 2.5. Classification Algorithm

**Figure 4:**
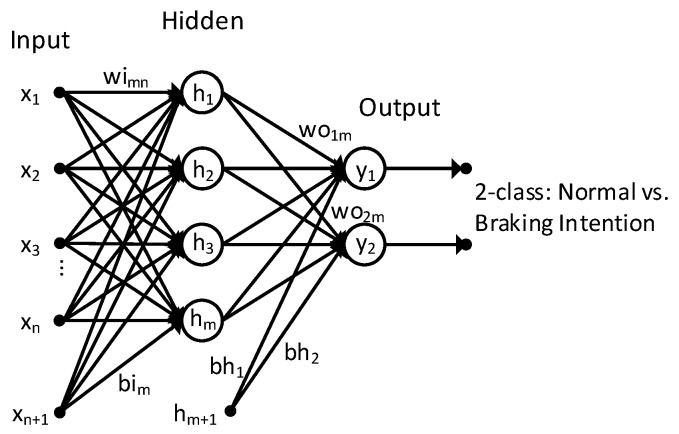
Neural-network structure for the two-class classifier (normal driving vs. braking intention).

In this study, a multilayer perceptron neural network is used as the classifier. We select neural network as the classifier because it can approximate any nonlinear function and can thus replace popular machine-learning approaches such as linear discriminant analysis (LDA) and support vector machines (SVM). Figure 4 shows the structure of the network that is used. The proposed neural network comprises three layers: input, hidden, and output. The size of the input layer of the network is equal to the size of the input features. For the PSD-based feature extractor, the feature covers five EEG bands (delta, alpha, theta, beta, and gamma), resulting in 40 inputs (5 bands × 8 channels). For the AR model feature extractor, the size of the feature is equal to the AR model order number multiplied by the number of EEG channels (eight). As mentioned previously, different AR model order numbers are investigated to obtain the best classification performance. Thus, the size of the input layer of the network is equal to 32, 40, 48, 56, 64, 72, 80, 88, 96, 104, 112, 120, 128, 136, 144, 152, 160, and 168 corresponding to AR model order number of 3, 4, 5, 6, 7, 8, 9, 10, 11, 12, 13, 14, 15, 16, 17, 18, 19, and 20, respectively. One disadvantage of the multilayer perceptron is that it requires tuning the number of hidden neurons. Therefore, in this study, we also investigate the system performance with respect to the number of hidden neurons (10, 20, 50, 100, 500, and 1000) to obtain the best network configuration. The output layer comprises two neurons corresponding to two classes: normal driving and braking intention. Sigmoid is chosen as the activation function of the hidden neurons, and softmax is chosen as the activation function of the output neurons. The general form of the softmax function is:

(5) $$f_{j}\left(z\right)=\frac{e^{z_{j}}}{\sum_{k=1}^{K}e^{z_{k}}}\;,\;j=1,\ldots ,k$$

The function takes a real-valued input vector *z* and maps it to a vector of real values in the range (0,1). Thus, an input data point is predicted to belong to a class if its mapping output value obtains a higher probability than those for other classes. In conjunction with softmax output, cross-entropy is chosen as the loss function for evaluating the quality of the neural network. It is noted that the current study applied the subject-specific training procedure. Particularly, for each subject, we collect EEG data and train the neural network model. Then, the well-trained network is validated and tested with the new dataset from the same subject. The number of samples during training and validation are varied with different setting parameters of the model. For instance, with a 1000-ms pre-braking period and 125-ms step-size, the entire dataset consists of 1418 samples (i.e., 922 samples of normal driving and 496 samples of braking intention (62 execution braking events × 8 segmented windows (~1000 ms/125 ms))). All data samples in the dataset were mixed before being split into train and validation sets. The training set consists of ~1100 samples (4 folds) whereas the validation set consists of ~280 samples (1 fold) according to 5-fold cross-validation method. These results indicated the discriminative capability of the dataset into two groups “normal driving” and “braking intention”.

## 3. Results

### 3.1. EEG Features

**Figure 5:**
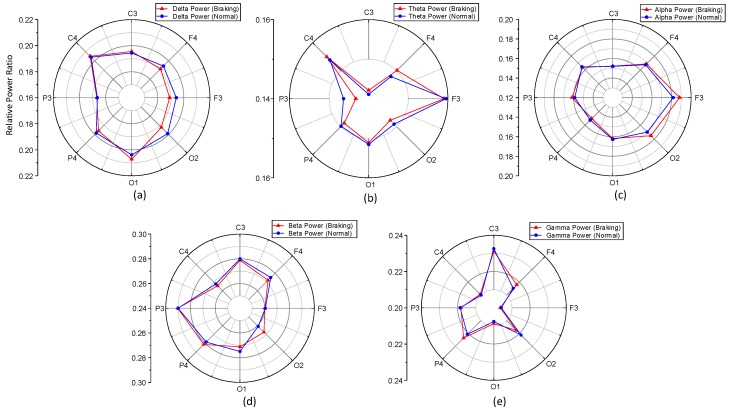
Comparison of the relative EEG power of eight channels between two situations: normal driving and braking intention: (**a**) delta; (**b**) theta; (**c**) alpha; (**d**) beta; and (**e**) gamma bands.

Figure 5 compares the PSD-based features of five EEG bands over eight channels between the normal driving and the emergency braking intention situations for subject 4. In general, the relative band powers of the five EEG bands were significantly or marginally different for many channels between these two situations. For instance, the relative power of the delta band was significantly different for channel F3 (*p* < 0.05) (Figure 5a). In the case of the theta band, the largest difference in the relative power was observed for channel P3 (*p* < 0.05) (Figure 5b). A marginal difference in the relative power of the alpha band was observed for channels F3 and F4 (*p* = 0.07 and *p* = 0.06, respectively) (Figure 5c). The relative power of the beta band was significantly different for channel O2 (*p* < 0.05) (Figure 5d). Similarly, in the case of the gamma band, significant differences in the relative power were observed for channels F4 and O2 (*p* < 0.05) (Figure 5e).

### 3.2. IMU Features

IMU sensor data obtained over approximately 10 min of driving during the reaction test are shown in Figure 3a. Obviously, emergency braking events are clearly indicated by abrupt changes in the amplitude of the signals (both accelerometer and gyroscope sensors). There were no significant amplitude changes during normal driving (Figure 3a, bottom). The IMU features were automatically obtained by sliding the 1-s data window along the sensor data. As shown in Figure 3b, if the standard deviation of the segmented data exceeded the predefined threshold, the sample was labeled as “braking intention.” Meanwhile, if the standard deviation was lower than the threshold, the sample was labeled as “normal driving.” The standard-deviation thresholds for the gyroscope and accelerometer sensors were set as 120 and 12, respectively. Figure 3c shows the distribution of the time interval between two consecutive braking events generated by the simulator software. The average value was calculated to be approximately 23 s. This is reasonable because the interval is long enough for the subject to calm down after the emergency situation. The average reaction time of the drivers to the emergency situation was calculated to be 0.88 s (Figure 3d). The driving simulator calculated this value by measuring the time interval between the emergency-stimulus onset and the onset of the executed braking event.

### 3.3. System Performance

**Figure 6:**
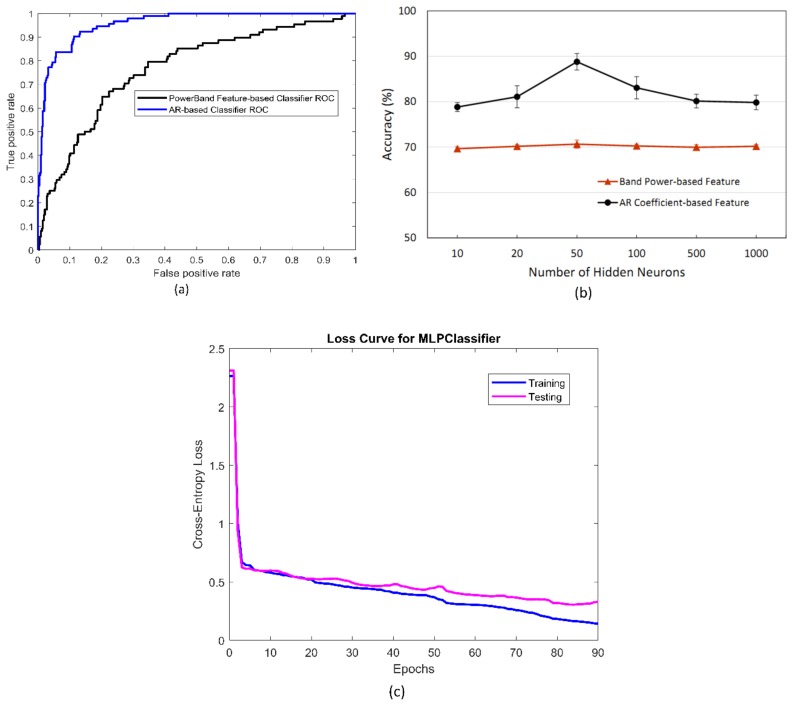
Performance comparison of the model between band power-based and autoregressive (AR)-based features for subject 4: (**a**) receiver operating characteristic (ROC) curves for the band power-based and AR-based classifiers; (**b**) system accuracy with respect to the number of hidden neurons in the artificial neural network (ANN) model; (**c**) cross-entropy loss during training and testing of the AR model-based classifier.

Figure 6 compares the system performance between the band power-based and AR-based features. The performance was compared based on several metrics, including receiver operating characteristic (ROC) curve, accuracy, sensitivity, and specificity. Accuracy, sensitivity, and specificity are calculated based on the confusion matrix as follows:

(6) $$Accuracy=\;\frac{TP+TN}{TP+TN+FP+FN}\;Sensitivity=\;\frac{TP}{TP+FN}\;Specificity=\;\frac{TN}{TN+FP}$$

where TP, TN, FP, and FN represent true positive, true negative, false positive, and false negative, respectively. According to the results, the ROC for the AR-based classifier indicated better performance than that for the band power-based classifier owing to its larger area under the curve (AUC) (i.e., 0.96 vs. 0.78) (Figure 6a). Moreover, the band power-based model achieved a classification accuracy of 67%, a sensitivity of 51%, and a specificity of 78% during validation. On the other hand, a classification accuracy of 90.8%, a sensitivity of 88.4%, and a specificity of 91.8% were achieved with the AR model-based classifier. These results were obtained under five-fold cross-validation with 50 neurons in the hidden layer, 0.125-s step size, and 1000-ms pre-braking period. A comparison of the system accuracy between the two aforementioned types of features with respect to the number of neurons in the hidden layer is shown in Figure 6b. In all the cases, the average accuracy during the five-fold cross-validation was higher for the AR-based classifier than for the band power-based classifier. The model exhibited the highest accuracy with a 50-neuron hidden layer. Therefore, a network configuration with 50 neurons in the hidden layer was employed for analysis, as described later. Owing to its better performance, the AR-based feature was selected for the system configuration. Cross-entropy loss curves during training and validation of the neural network classifier utilizing AR model-based feature is shown in Figure 6c. The best cross-entropy loss during training was achieved at 0.14 after 90 iterations resulting in 92.6% accuracy. Accordingly, this well-trained model yielded a testing accuracy of 90.8% with corresponding cross-entropy loss of 0.35.

**Figure 7:**
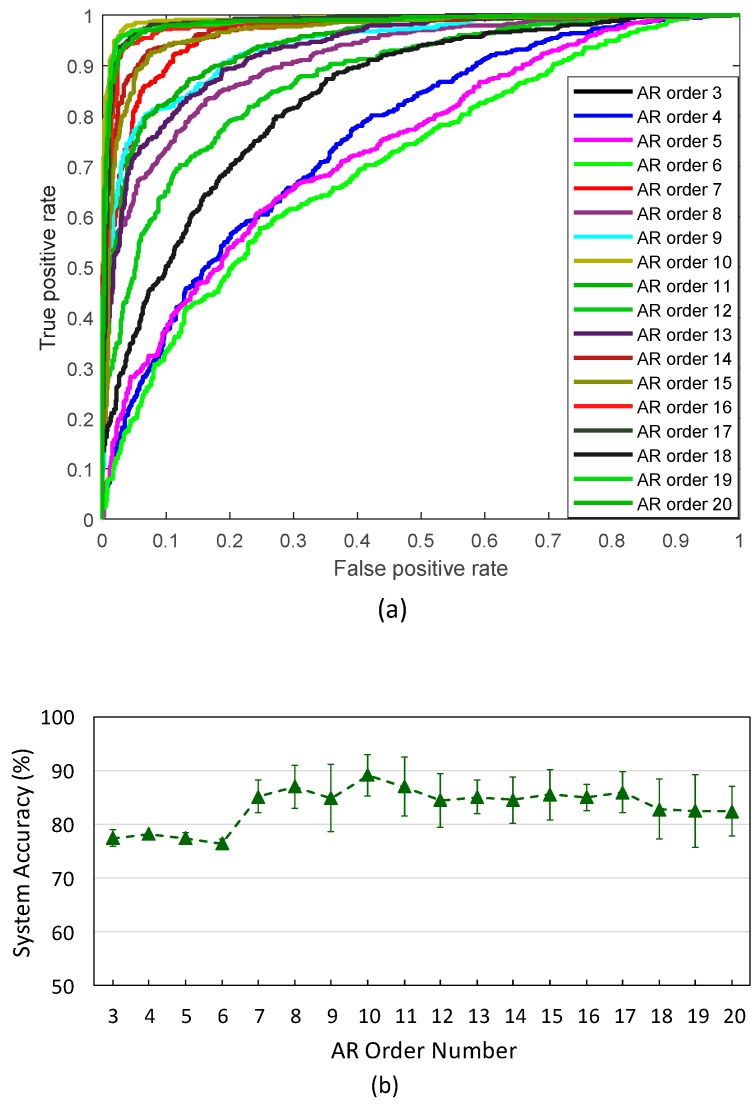
System accuracy with respect to the AR order number: (**a**) ROC curves for different AR classifiers with different AR order numbers; (**b**) average system accuracies during five runs of the neural-network model.

As mentioned previously, an important step in AR modeling is selecting an appropriate AR order number. If the AR model order is too low, the signal can be lost. Meanwhile, if the order is too high, a large amount of noise is captured [28]. Model orders of 5 and 10 appear to best track the dynamics of the low-frequency band of EEG signals [29]. Figure 7 compares the system performance among different AR orders (from 3 to 20) (step size was set to 0.125 s). Typical ROC curves for different classifiers utilizing different AR orders are shown in Figure 7a. According to these results, the highest performance of the model was achieved with AR orders of 10, 11, 12, 14, 15, 16, 17, and 19 (AUC > 0.94). The AR orders of 3, 4, 5, and 6 yielded the worst model performance. A comparison of the system accuracy among these different AR orders during five-fold cross-validation is presented in Figure 7b. The results showed that the model achieved an accuracy above 80% with higher AR orders (from 7 to 20). However, in comparison with the lower AR orders, the five-fold cross-validation results indicated a greater variation of the system accuracy in cases of high AR orders. To achieve high accuracy of the system, the order of the AR feature extractor was selected as 10.

**Figure 8:**
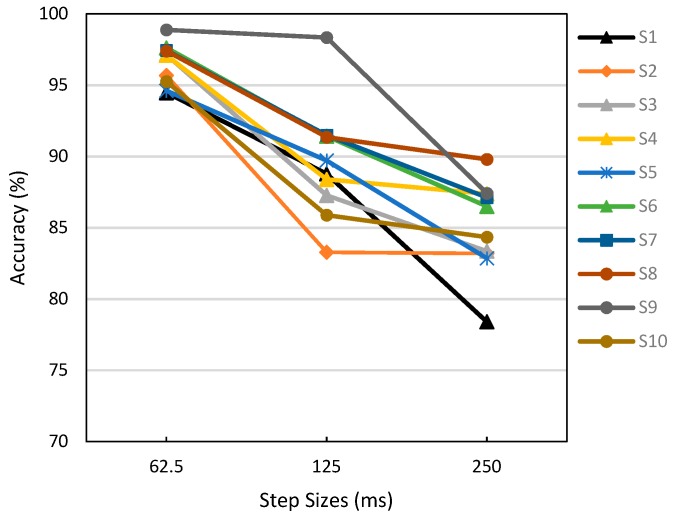
System accuracy with respect to the analysis step size for 10 subjects.

Figure 8 shows the effect of the step size on the system accuracy. We estimated the computational time of the system for a single cycle of feed-forward computation (prediction) to be approximately 48 ms. Hence, three different step sizes including 62.5 ms (8 samples), 125 ms (16 samples), and 250 ms (32 samples) were investigated to obtain the best system performance with regard to the detection accuracy and detectable time. For all subjects, the system exhibited the best accuracy (approximately 95%) with a short step size of 62.5 ms. A significant accuracy reduction occurred when the step size increased for most subjects, with the exception of subject 9. There was no significant difference in the accuracy between the step sizes of 125 and 250 ms, except for subjects 1 and 9. Thus, the step size of 62.5 ms was selected for the system.

**Figure 9:**
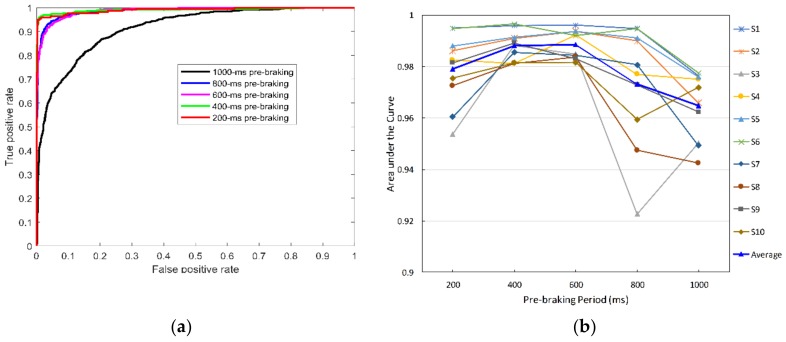
Labeling the braking intention samples within different pre-braking periods: (**a**) ROC curves; (**b**) AUC of the AR feature-based classifiers with respect to pre-braking period for 10 subjects.

As mentioned in Section 2.4, one of the hidden variables in the evaluation of the system is the neurophysiological response time to the emergency event after the onset of the emergency stimulus. To investigate this parameter, we evaluated the system with different pre-braking periods, which were labeled as braking-intention samples. Figure 9a shows a comparison of the classification performance of the model among these cases, based on the AUC. Obviously, the model exhibited the highest performance with pre-braking periods of 200 and 400 ms, followed by 600 and 800 ms (AUC > 0.95). The lowest performance of the model occurred in the case of a 1000-ms pre-braking period (AUC = 0.86). Figure 9b shows the system performance (indicated by the average AUC) with respect to the pre-braking period for 10 subjects. The data were obtained using five-fold cross-validation. The system exhibited good performance with the 400- and 600-ms pre-braking periods for most subjects. The largest AUC of 0.989 was obtained with the 600-ms pre-braking period. The results indicated that the neurophysiological response occurred approximately 600 ms before the onset of the executed braking event.

**Figure 10:**
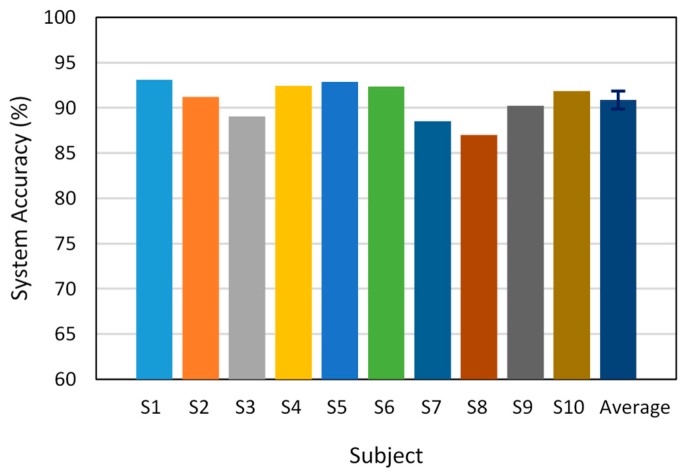
Accuracy of the proposed system in online testing for 10 subjects.

The model evaluation via online testing during 10 minutes of driving is shown in Figure 10. These results were obtained with the best configuration of the model based on the offline evaluation: an AR feature extractor with order 10, 50 neurons in the hidden layer, a 600-ms pre-braking period, and a step size of 62.5 ms. Accordingly, the system exhibited an accuracy above 90% for subjects 1, 2, 4, 5, 6, 9, and 10. The lowest accuracy occurred in the case of subject 8 (~87%). The average accuracy for the 10 subjects was 90.8%.

## 4. Discussion

In this paper, we developed a novel system using EEG signals for detecting the emergency braking intention during simulated driving. The system consists of a custom-designed EEG headset with eight channels integrated with an IMU sensor. It was completely fabricated via 3-D printing using a flexible material and was comfortable for users. The detection kernel was based on machine learning using a multilayer perceptron neural network. The feature extraction was based on AR model and EEG band power approaches. It turned out that the AR-based feature outperformed the band power-based feature with regard to classification accuracy.

In machine learning, labeling the data prior to the training process plays an important and essential role which partially decides the system performance. Compared with [30], a new method for accurately and reliably labeling the training data based on motion-sensing was introduced in this work. Generally, compared with previous studies [18,22], several aspects have been considered to make the proposed system more robust and reliable. For instance, the offline training procedure that was identical (i.e., windowing analysis, labeling process) to the online testing was conducted. Moreover, the training dataset was sufficiently large for the training process. Detailed analyses of several aspects, such as the effect of the step size in a real-time detection system and the most likely physiological response after the onset of an emergency braking situation were performed.

The results indicated that the combination of the AR feature extractor and the neural-network classifier achieved better performance in comparison with that utilizing the EEG band power-based feature. Experimental results for 10 subjects showed that on average, the proposed system could detect the emergency braking intention approximately 600 ms before the onset of the executed braking event, with an accuracy of 91%. The highest detection accuracy of the proposed system was obtained with a 600-ms pre-braking period highlighting the most likely physiological response after the onset of the emergency braking situation. On the other hand, with a 1000-ms pre-braking period, the system yielded the lowest detection accuracy. Since the average reaction time was measured (by the simulated software) to be 880 ms after the onset of the emergency stimulus, the low accuracy of the system, in this case, might have resulted from overlapping windows between “braking intention” samples and the “normal driving” samples (before the stimulus).

With this study, we highlighted the feasibility of capturing neural responses to the emergency situation. Specifically, the braking intention of the driver during facing an emergency situation can be recognized. The finding can be also applied in other applications. For instance, instead of using motor imagery (MI) [31] to control the wheelchair to avoid the obstacle, we can detect the neural activities response to facing the obstacle for naturally change the wheelchair direction. In fact, different level of attention of the drivers results in different level of surprising or fear during facing an emergency situation. Therefore, according to [32], it is reasonable and necessary to explore the neural responses to emergency situations or threats under different level of attention.

Besides, the current study had several limitations need to be considered in the next steps. First, the tests were performed using a driving simulator. Hence, external effects such as vibrations caused by the road, engine noise, and the vehicle and the light intensity in the car were not considered. Second, more driving scenarios need to be tested, e.g., urban vs. rural areas. In fact, driving at night is more dangerous than driving in the day owing to the lack of sleep or limited visibility of drivers. Consequently, an emergency situation would affect the brain more significantly during nighttime driving than during daytime driving. Hence, the system performance should be compared between daytime and nighttime driving. Additionally, the proposed system should be tested in a real driving environment prior to being employed in real-life applications.

## 5. Conclusions

In summary, our proposed system can be used as an alternative to the conventional braking method (i.e., foot braking) by sending an early braking signal to the vehicle. Moreover, it is also useful for disabled people who have difficulty braking during driving. The results indicated the feasibility and practicality of implementing a reliable braking-assistance system for vehicles.
